# Supplementary material for: Investigating Differential Expressed Genes of Limosilactobacillus reuteri LR08 Regulated by Soybean Protein and Peptides
Source: Foods. 2022 Apr 26;11(9):1251. doi: 10.3390/foods11091251 (PMC9105380; doi:10.3390/foods11091251)
Supplement: Supplementary file 1 [file foods-11-01251-s001.zip › foods-1662907-supplementary.pdf]

(A)

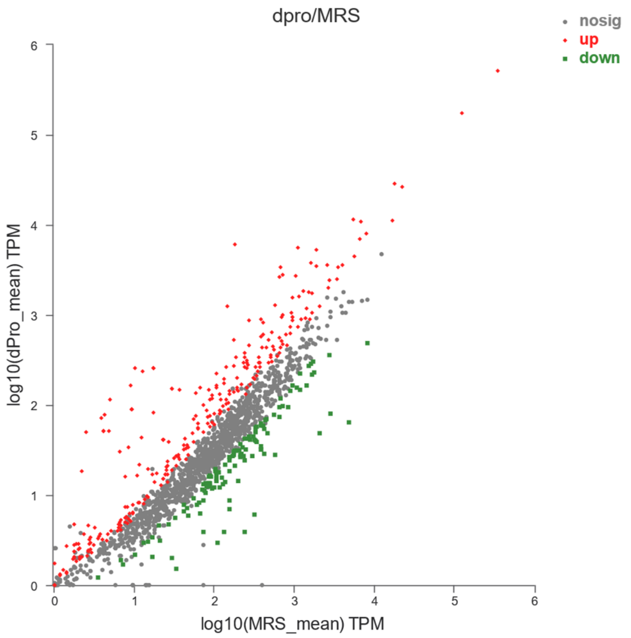
dpro/MRS
nosig
up
down
log10(dPro_mean) TPM
log10(MRS_mean) TPM
0
1
2
3
4
5
6

(B)

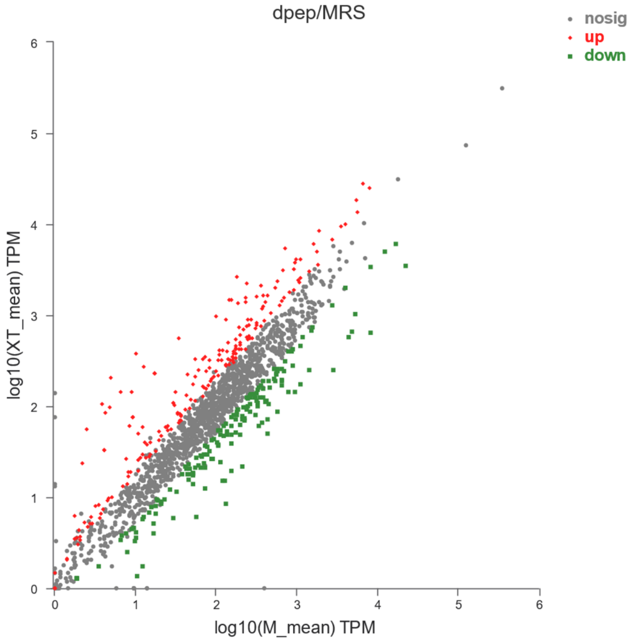
dpep/MRS
nosig
up
down
log10(XT_mean) TPM
log10(M_mean) TPM
0
1
2
3
4
5
6

(C)

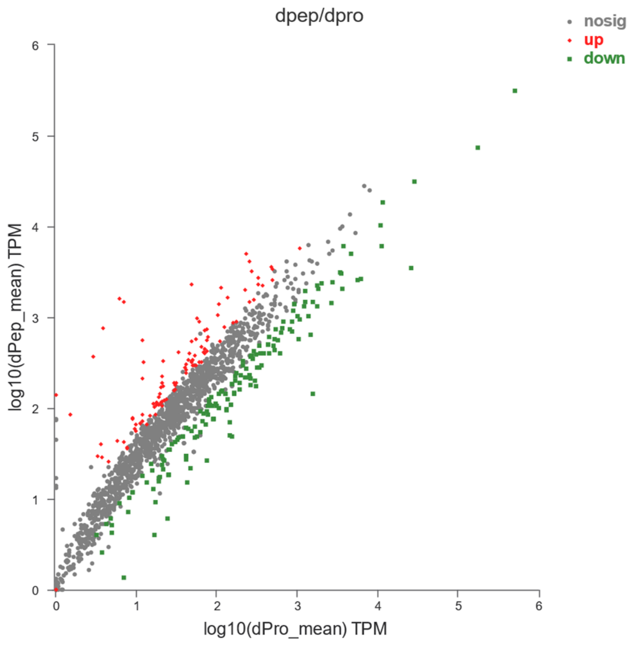

Figure S1. The scatter map of DEGs under different comparisons (n=3), (A) MRS/dpep; (B) MRS/dpro; (C) dpro/dpep. Dots in red, green and grey indicate significantly up-regulated genes, down-regulated genes, and non-significantly different genes. After mapping all genes, points with deviation indicate the diversity of gene expression between the two samples.

Table S1. Primer sequences of critical DEGs and reference genes

Primer	Sequence
***purN* -F**	5'- ACCGAATTATTAACCTTCATCCA -3'
***purN* -R**	5'- CTGTCTGTGCTTGCTGAT -3'
***purF* -F**	5'- GATATGGTGATTGGAGTA -3'
***purF* -R**	5'- TCTTGAGTTGGTTGAATA -3'
***purM* -F**	5'- ACGATTCTGACTCCTACTA -3'
***purM* -R**	5'- ATCAATCCTCCACCAGTA -3'
***accD*-F**	5'- TCAAGCAGTAGTATTATTC -3'
***accD*-R**	5'- GTTAAGACCACAATGTAT -3'
***fabI*-F**	5'- GCTATTCCTAACTATAATGT -3'
***fabI*-R**	5'- ATACCTGTAACTGCTAAT -3'
***glnA*-F**	5'- TATTAACGGTTCAGGAAT -3'
***glnA*-R**	5'- TATAGGAGTTGACGATTG -3'
***accA* -F**	5'- AGTCAACTTGCTATTCCA -3'
***accA* -R**	5'- TTCTAACATCCATACCTCAT -3'

N134_RS06565 **-F**	5'- AAGCCATTAAGGAAGTAGG -3'
N134_RS06565 **-R**	5'- CGGAAGTTGAACCAGTAA -3'
N134_RS00710**-F**	5'- ATCTGACAACTAAGCAACATAT-3'
N134_RS00710**-R**	5'- CTTCTAACCGTTCCAATACC-3'
rpmA**-F**	5'- TCAATCATCTACCGTCAA-3'
rpmA**-R**	5'- CCTTAGCGTATAATGTATCG-3'
metC **-F**	5'- TGTAGTTGGTAGTGATGT-3'
metC **-R**	5'- GTAAGAGCGGATTAGTTG-3'
tcyB**-F**	5'- CGATTCCGTTAGCGATTA-3'
tcyB**-R**	5'- ATTCTCAGATATGGCAGTC-3'
tcyC **-F**	5'- TTCGCTCGCTTAACTTAT-3'
tcyC **-R**	5'- GTTGGTCCTTCAATAATGTT-3'
opuC **-F**	5'- CAAGATTATGGAACAAGGA-3'
opuC **-R**	5'- TTAACTTCGCATCAACTT-3'
artR **-F**	5'- CTCTTCATCAACCAATAATC-3'
artR **-R**	5'- GGATAATCAGCACTCATT-3'
glnH **-F**	5'- TGATTCCTTCGTTAGTTA-3'
glnH **-R**	5'- GGTAATGATGATGATATACA-3'
Lpla16rsRNA **-F**	5'- GCTCGTGTCGTGAGATGTT-3'
Lpla16rsRNA **-R**	5'-TGTAGCCCAGGTCATAAGG-3'

Table S2. Overall descriptions of critical DEGs

Gene Name	Gene Function Description	GO Function	KEGG Pathway Description	Fold Change			RT-qPCR Verification		
				MRS/dpep	MRS/dpro	dpro/dpep	MRS/dpep	MRS/dpro	dpro/dpep
purN	Phosphoribose glycinamide formyltransferase	Biological process: synthesis process of 'denovo' IMP Molecular function: activity of phosphoribosyl glycinamide formyltransferase	purine metabolism, a "carbon pool" of folic acid	36.83	76.31	-1.96	526.83	85.29	3.09
purF	Amido phosphoribosyltransferas e	Biological process: glutamine metabolism process, purine nucleoside biosynthesis process, "denovo" IMP biosynthesis process and nucleoside metabolism process Molecular function: amido phosphoribosyltransferas e activity, Magnesium ion binding	metabolism of alanine, aspartic acid and glutamic acid,purine metabolism	34.13	90.90	-2.56	542.59	143.14	3.81

purM	Phosphoribose formyl glycinamide ring ligase	Cell component: cytoplasm Molecular function: activity of phosphoribosyl formyl glycinamide ring ligase, ATP binding Biological process: biosynthesis process of ' denovo' IMP	purine metabolism	24.11	71.68	-2.86	1140.87	305.09	3.76
accD	Acetyl coenzyme a carboxylase carboxylase subunit β	Biological process: biosynthesis process of fatty acids Cell component：acetyl coenzyme a carboxylase complex Molecular function: carboxyl or carbamoyl transferase activity, zinc ion binding, acetyl-CoA carboxylase activity, ATP binding Biological process: biosynthesis of malonyl coenzyme A	propionate metabolism of carbon fixation pathway in prokaryotes, fatty acid biosynthesis, pyruvate metabolism	3.69	-11.11	66.19	65.75	4.34	15.20

fabI	Enoyl -ACP reductase	Biological process: fatty acid biosynthesis process Molecular function: enoyl ACP reductase (NADH) activity, enoyl ACP reductase activity	biotin metabolism, fatty acid biosynthesis, biosynthesis of prodigiosin	10.27	-7.69	82.86	29.94	1.13	26.48
glnA	Glutamate ammonia ligase type I	Molecular function: glutamate-ammonia ligase activity, ATP binding	metabolism of glyoxylate and dicarboxylate arginine biosynthesis nitrogen metabolism, alanine, aspartic acid and glutamic acid metabolism	-5.88	-1.18	-4.55	5.18	7.41	-1.43
accA	Carboxytransferase subunit of acetyl-CoA carboxylase	Biological process: biosynthesis of fatty acids Cell component: acetyl coenzyme a carboxylase complex Molecular function: transferase activity,	carbon fixation pathway in prokaryotes, propionic acid metabolism, fatty acid biosynthesis,	5.49	-20.0	115.24	32.37	1.52	21.33

		acetyl coenzyme a carboxylase activity	pyruvate metabolism						
N134_RS06565	Glutamate -tRNA ligase	Cell component:cytoplasm Biological process: glutamyl -tRNA aminoacylation Molecular function: tRNA binding, glutamate -tRNA ligase activity, zinc ion binding, ATP binding	biosynthesis of aminoacyl-tRNA	-4.35	-1.56	-2.56	1.77	2.76	-1.54
N134_RS00710	phosphoribosyl aminoimidazole succinyl carboxamide synthase	Molecular function: activity of phosphoribosyl aminoimidazole succinyl carboxamide synthase Biological process: biosynthesis process of cobalamin, “abinitio” IMP biosynthesis process Molecular function: ATP binding	purine metabolism	23.51	35.66	-1.45	583.40	214.46	2.72
rpmA	50S ribosomal protein L27	Molecular function: structural components of ribosomes	ribosome	-5.56	-2.86	-1.79	1.17	1.60	1.37

		Biological process: translation Cell component: ribosome							
tcyB	Amino acid ABC transporter permease	Biological process: transport of nitrogen compounds Cell component: ATP binding cassette (ABC) transporter complex Molecular function: transmembrane transporter activity	ABC transshipment	7.41	3.69	2.13	34.12	16.70	2.05
metC	PLP-dependent transferase	Molecular function: c0atalytic activity, lyase activity, L-cysteine dehydrogenase activity, L-cystine L-cysteine lyase (deamination) Biological process: transfer sulfur Molecular function: transferase activity, pyridoxal phosphate binding, cystathionine γ-synthase activity,	metabolism of selenium compounds, metabolism of cysteine and methionine	10.19	4.45	2.43	43.25	23.29	1.86

		cystathionine γ -synthase activity							
tcyC	Amino acid ABC transporter ATP binding protein	Molecular function: amino acid transport ATPase activity, ATP binding, ATPase activity	ABC transshipment	7.01	3.33	1.15	25.11	14.66	1.72
opuC	Glycine/betaine ABC transporter substrate binding protein	Cell component: ATP binding cassette (ABC) transporter complex Molecular function: transmembrane transporter activity Biological process: transport of nitrogen compounds	ABC transshipment	6.48	1.33	5.18	11.33	1.95	5.83
artR	ABC transporter substrate binding protein/osmotic enzyme	Cell component: ATP binding cassette (ABC) transporter complex Molecular function: transmembrane transporter activity	ABC transshipment	-9.09	-3.57	-2.50	-3.70	3.93	-14.29

glnH	ABC transporter substrate binding protein/osmotic enzyme	Biological process: transport of nitrogen compounds Cell component: ATP binding cassette (ABC) transporter complex Molecular function: ligand gate ion channel activity	ABC transshipment	-8.33	1.01	-8.33	4.62	5.81	-1.25

(n=3)
